# Structural Equation Modeling of Latent Growth Curves of Weight Gain among Treated Tuberculosis Patients

**DOI:** 10.1371/journal.pone.0091152

**Published:** 2014-03-11

**Authors:** Mahalingam Vasantha, Perumal Venkatesan

**Affiliations:** Department of Statistics, National Institute for Research in Tuberculosis (formerly Tuberculosis Research Centre), Indian Council of Medical Research, Chennai, India; Fundació Institut d'Investigació en Ciències de la Salut Germans Trias i Pujol. Universitat Autònoma de Barcelona. CIBERES, Spain

## Abstract

Tuberculosis still remains a major public health problem even though it is treatable and curable. Weight gain measurement during anti tuberculosis (TB) treatment period is an important component to assess the progress of TB patients. In this study, Latent Growth Models (LGMs) were implemented in a longitudinal design to predict the change in weight of TB patients who were given three different regimens under randomized controlled clinical trial for anti-TB treatment. Linear and Quadratic LGMs were fitted using M*plus* software. The age, sex and treatment response of the TB patients were used as time invariant independent variables of the growth trajectories. The quadratic trend was found to be better in explaining the changes in weight without grouping than the quadratic model for three group comparisons. A significant increase in the change of weight over time was identified while a significant quadratic effect indicated that weights were sustained over time. The growth rate was similar in both the groups. The treament response had significant association with the growth rate of weight scores of the patients.

## Introduction

India remains one of the highest TB burden countries with an estimate of 2.2 million cases out of global incidence of 8.7 million cases and 300, 000 deaths out of 1.4 million global TB death in 2011 [1]. The Revised National Tuberculosis Control Programme (RNTCP) was implemented in India in 1993 using DOTS recommended by World Health Organisation, where the patients received treatment thrice weekly. Under the RNTCP, patients with new smear-positive pulmonary TB are treated with a 6-month thrice-weekly regimen starting with 2 months of isoniazid (H), rifampicin (R), pyrazinamide (Z) and ethambutol (E), followed by 4 months of H and R (2H3R3Z3E3/4H3R3) [2]. The emergence and spread of MultiDrug Resistant (MDR) tuberculosis and extensively drug resistant (XDR) tuberculosis threaten global TB control measures. In order to decrease the drug resistance induced TB infections, WHO in 2007, and subsequently in 2010, advised daily treatment as the preferred drug regimen for treating TB patients [3].

In the current study, a longitudinal design was used where TB patients randomly received three different regimens at National Institute for Research in Tuberculosis, Chennai, India. The three regimens were selected for this trial: a completely unsupervised daily regimen of 8 months' duration with EHRZ during the initial 2 months followed by EH for the next 6 months' duration; two twice-weekly regimens of 6 months' duration: one with the same four drugs during initial 2 months, followed by three drugs (HER) for the next four months and third regimen similar to the second except that it did not contain E. The latter two regimens were either completely or partially supervised [4]. The benefits of these regimens are mentioned in various studies [4], [5], [6], [7].

The weight gain of the patients at various time points during anti TB treatment period is an important component to assess the progress of the patients as reported by numerous studies [8], [9], [10], [11]. A study from Peru used Generalized Estimating Equations to assess the relationship between the treatment outcome and the trend of body weight of the patients over time [12]. To our knowledge, apart from the Peru study, longitudinal analysis of weight gain in TB patients over time during the treatment period was not reported. The use of latent growth modeling to identify the trend of TB patients' weight was not studied so far. Latent growth model (LGM) is used in structural equation modeling (SEM) to estimate growth trajectory over time. SEM advances basic longitudinal analysis of data to include latent variable growth over time while modeling both individual and group changes using slopes and intercepts [13], [14], [15]. The objective of this study is to analyze growth trajectory of weight scores of these patients using LGM.

## Materials and Methods

### Randomised controlled clinical trial TB data

A total of 600 TB patients who were treated with anti-TB treatment under a randomised controlled clinical trial at National Institute for Research in Tuberculosis, Chennai were considered for this study. The details of design and other particulars can be found elsewhere [4]. The patients who aged 12 years or more were residents of the metropolis of Chennai in south India, with at least two sputum smears positive for acid-fast bacilli. These patients were allocated to three treatment groups consists of one 8 months and 2 six months with first line anti-TB drugs. Regimen 1 (Reg. 1): 2EHRZ_7_/6EH_7_: daily chemotherapy of 8 months' duration, given completely unsupervised, Reg. II: 2EHRZ_2_/4EHR_2_: 6 month twice weekly regimen, Reg. III: 2HRZ_2_/4HR_2_: similar to regimen 2 without ethambutol (E). The study was approved by institutional ethics committee and informed consent was obtained from each patient before enrolling into the study. The progress of each patient was closely monitored during chemotherapy. The weights were recorded in monthly interval for a period of six months.

### Participants

**Figure 1:**
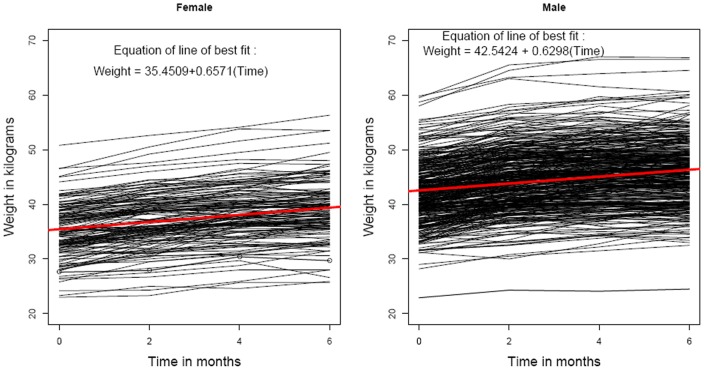
Scatter line diagram of weight of TB patients (in kilograms) versus time (in months) according to gender. Legend: Black lines - weight of TB patients. Red line - line of linear regression.

**Figure 2:**
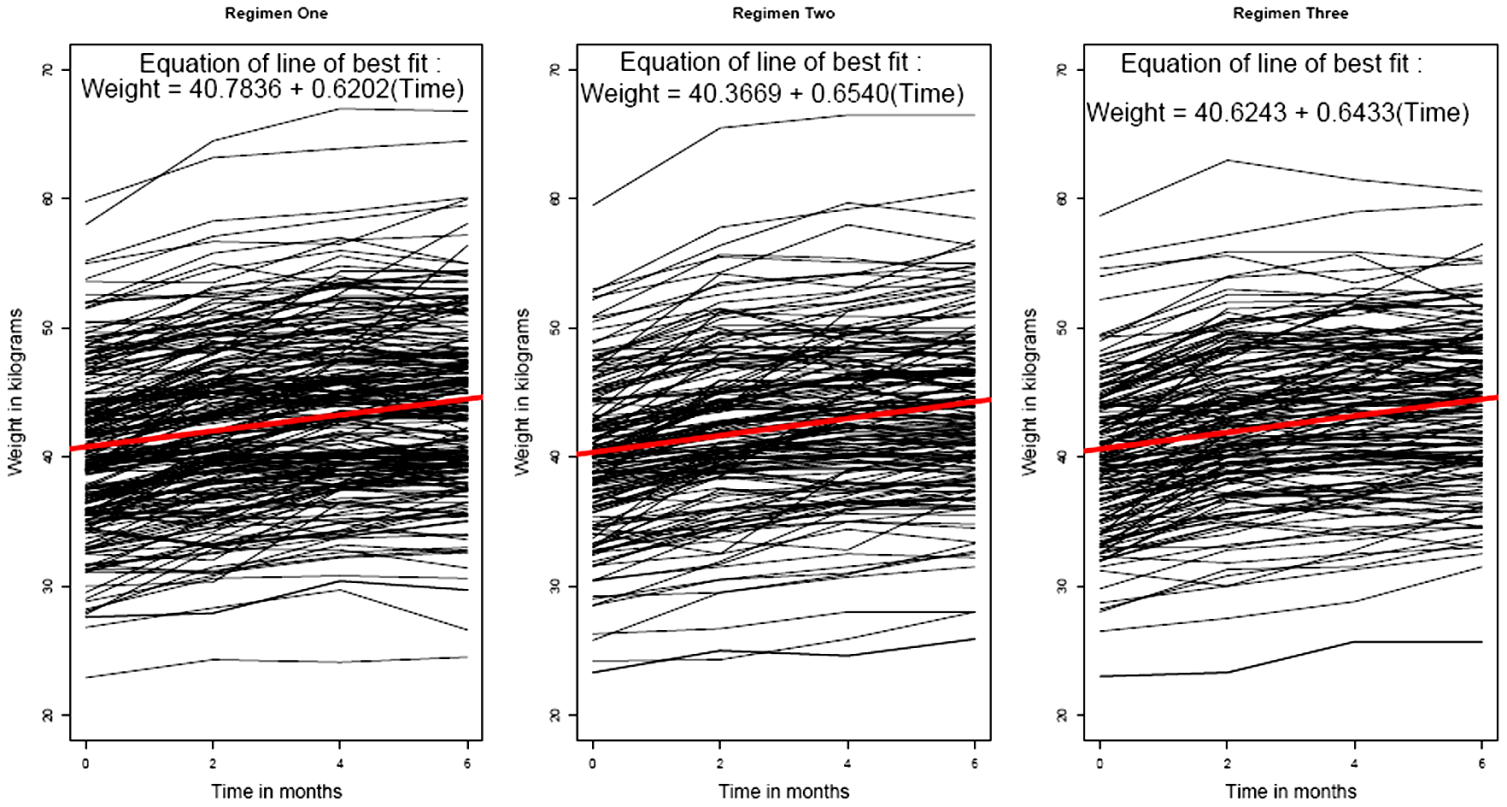
Scatter line diagram of weight of TB patients (in kilograms) versus time (in months) according to regimen. Legend: Black lines - weight of TB patients. Red line - line of linear regression.

**Figure 3:**
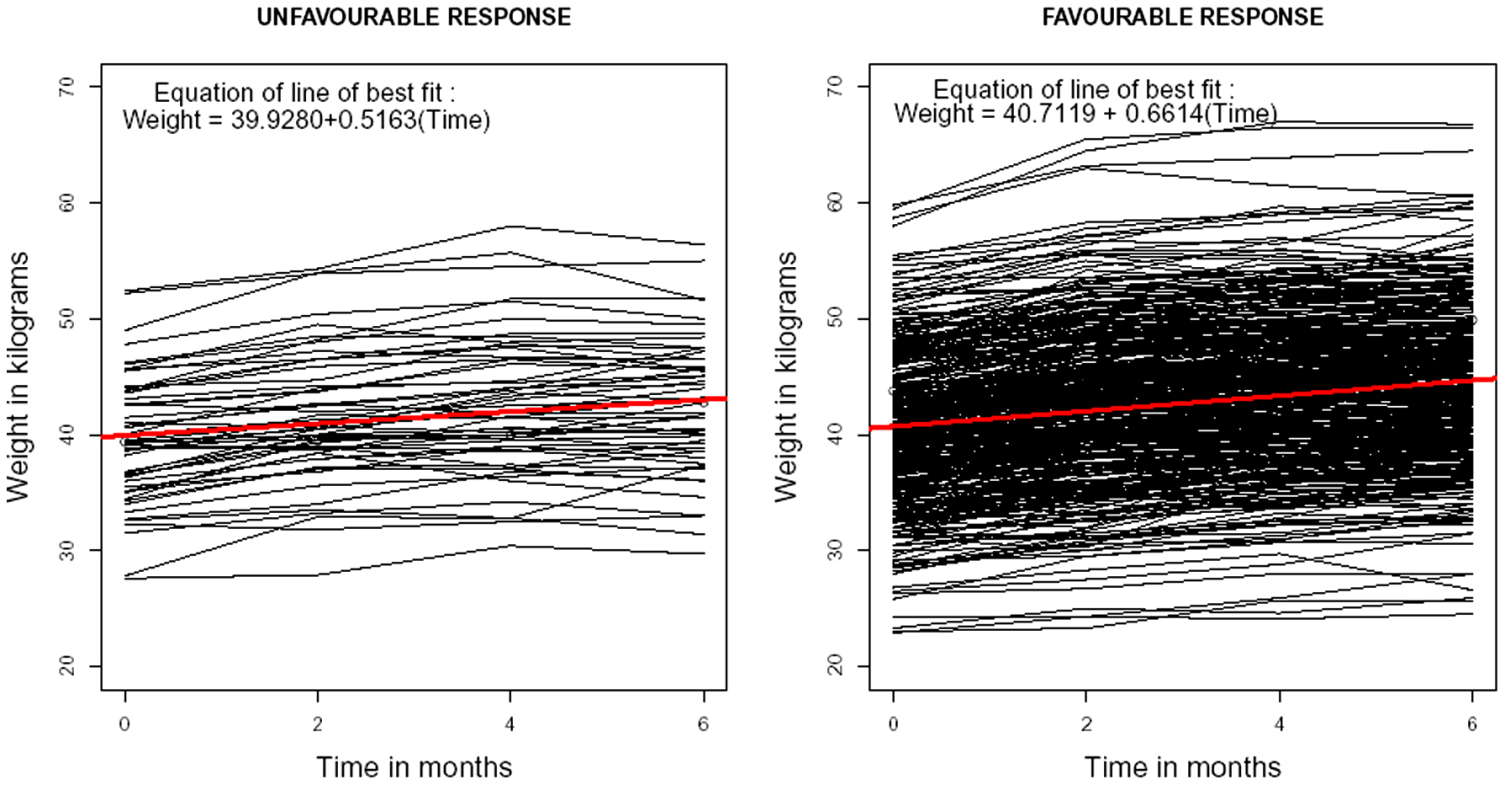
Scatter line diagram of weight of TB patients (in kilograms) versus time (in months) according to response status. Legend: Black lines - weight of TB patients. Red line - line of linear regression.

Out of 600 TB patients, 109 were excluded from the analysis: 69 cases due to non availability or missing values and 40 who had missed 20% or more of their chemotherapy. At the initiation of treatment, socio demographic profile of the 491 patients, were: 350 (71.3%) were males, the mean age was 30.8 years (range: 12–75 years) with mean weight of 40.1 kg (range: 22.9–59.8 kg). Of the 491 patients, 436 (88.8%) (Reg. I: 190/206 Reg. II: 132/142 and Reg. III: 114/143) had favourable response and 55 (11.2%) had unfavourable response (including doubtful response) at the end of treatment. The distributions of basic characteristics of the TB patients according to regimen are given in Table 1. The scatter line diagrams according to gender, regimen and outcome were given in Figures 1, 2 and 3 respectively.

**Table 1:** Base line characteristic of the TB patients.

Regimen	Sex		Age in years		Weight in kgs	
	Male	Female	Mean	Range	Mean	Range
I	147 (71.4%)	59 (28.6%)	31.1	12–56	40.3	22.9–59.8
II	93 (65.5%)	49 (34.5%)	30.9	14–55	39.9	23.3–59.5
III	110 (76.9%)	33 (21.3%)	30.1	12–75	39.9	23.0–58.7
Total	350 (71.3%)	141 (28.7%)	30.8	12–75	40.1	22.9–59.8

### Statistical methods

Unconditional LGMs were fitted to describe the growth trajectories of weight scores of the TB patients during the treatment period. Subsequently, to study the changes due to sex, age and response at the end of the treatment of the TB patients, these factors were added in conditional LGMs as time invariant covariates. Furthermore, the trend of weight measurements of the patients among the three regimen groups was compared using multiple group LGMs. LGMs were fitted using M*plus* version 7.0 for windows by method of Bootstrap [16].

The observed repeated measures wt0, wt2, wt4 and wt6 are the weights of the patients measured in kilograms from initial stage of treatment to sixth month at an interval of two months time period. For the initial iteration, the slopes were coded as 0, 2, 4 and 6 to establish linear trend [17]. The first factor loading assumed to be zero in order to find out mean value of initial time point [18]. The intercepts were coded as 1 to indicate means for different months. The following factor loading was used to estimate the quadratic equation model with origin fixed at the initial stage of treatment [19]. 
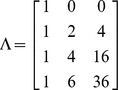



Our analysis using LGM was powerful in determining the response of anti TB treatment using weight scores as the measurement scale.

### Model fit and comparison

The most widely used test for checking global model fit is chi-square test that dependent on the sample size of the study population [20]. It rejects reasonable models if the sample size is large while it fails to reject poor models if the sample is rather small. The three other types of fit indices to assess the fit of a model are comparative fit indices, absolute indices and information theoretic indices [21]. Comparative indices compare the target model with fit of the base line model. For example, Comparative Fit Index (CFI) and Tucker-Lewis index (TLI), CFI and TLI range from 0 to 1. The TLI attempts to correct for complexity of the model but it is sensitive to a small sample size. Root Mean Square Error Approximation (RMSEA) is a measure of overall goodness-of-fit and related to the residual in the model. The RMSEA is insensitive to sample size but it is sensitive to model complexity. The Aikake information criterion (AIC) [22] and Bayesian information criterion (BIC) [23] are used to compare the best fitting model by choosing the model with smallest AIC and BIC from the models. The adequate fit is defined as CFI>0.9, TLI>0.9, RMSEA<0.08 [24], [25]. The M*plus* commands used for analyses are included in the appendix S1.

## Results

**Figure 4:**
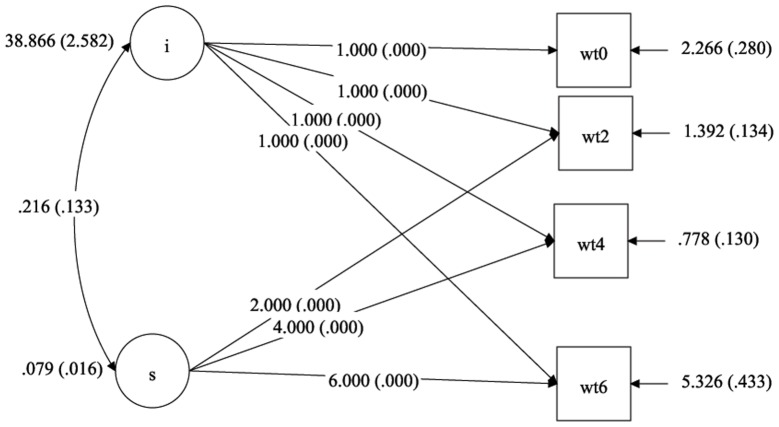
Linear latent growth model of weight change in TB patients over time. Legend: i - intercept of linear latent growth model of weight change in TB patients. s - slope of linear latent growth model of weight changes in TB patients. wt0 - weight of TB patients in kilograms at the initial stage of anti TB treatment. wt2 - weight of TB patients in kilograms at 2^nd^ month of anti TB treatment. wt4 - weight of TB patients in kilograms at 4^th^ month of anti TB treatment. wt6 - weight of TB patients in kilograms at 6^th^ month of anti TB treatment.

The unconditional Linear LGM was fitted for the weight scores of 491 TB patients and the fit indices were CFI  = 0.931, TLI  = 0.917 and RMSEA  = 0.329. The path diagram of linear LGM is given in Figure 4. The results of fit indices AIC, BIC, CFI, TLI and RMSEA are presented in Table 2. The change of weight scores of TB patients during treatment period was found to be nonlinear. When a basic quadratic LGM model of the weight scores was analysed for the 491 patients, the RMSEA value was reduced (CFI  = 0.992, TLI  = 0.987 and RMSEA  = 0.128) compared to linear LGMs when fixing the variance of quadratic effect as zero.

**Table 2:** Fit indices of linear LGM and Quadratic LGM.

Fit	Model		Model with	Model of 3 groups	
Indices	(n = 491)		bootstrap sample of 1000	comparisons with bootstrap sample of 1000	
	Unconditional Linear	Unconditional Quadratic	Conditional Quadratic	Unconditional Quadratic	Conditional Quadratic
AIC	9374.373	9142.080	8989.577	9043.458	8906.214
BIC	9412.141	9184.045	9056.720	9169.351	9107.643
CFI	0.931	0.992	0.991	0.985	0.986
TLI	0.971	0.987	0.983	0.978	0.974
RMSEA	0.329	0.128	0.088	0.173	0.110

In multiple group latent growth modeling (LGM), models are estimated simultaneously across the groups. Using multi-group LGM, the unconditional quadratic model was estimated for the groups using sample of 1000 by bootstrap method. The fit indices were CFI  = 0.985, TLI  = 0.978 and RMSEA  = 0.173. A significant increase in weight scores was observed in the all three groups whereas the increase in group three is higher than the group one and two (Group 1: Intercept  = 40.274 (S.E  = 0.458), p<0.001, slope  = 1.309 (S.E  = 0.068), p<0.001; quadratic effect  = −0.115, (S.E  = 0.012), p<0.001; Group 2: Intercept  = 40.031 (S.E  = 0.517), p<0.001, slope  = 1.343 (S.E  = 0.081), p<0.001; quadratic effect  = −0.112 (S.E  = 0.011), p<0.001; Group 3: Intercept  = 40.088 (S.E  = 0.496), p<0.001, slope  = 1.390 (S.E  = 0.087), p<0.001; quadratic effect  = −0.126 (S.E  = 0.013), p<0.001).

**Figure 5:**
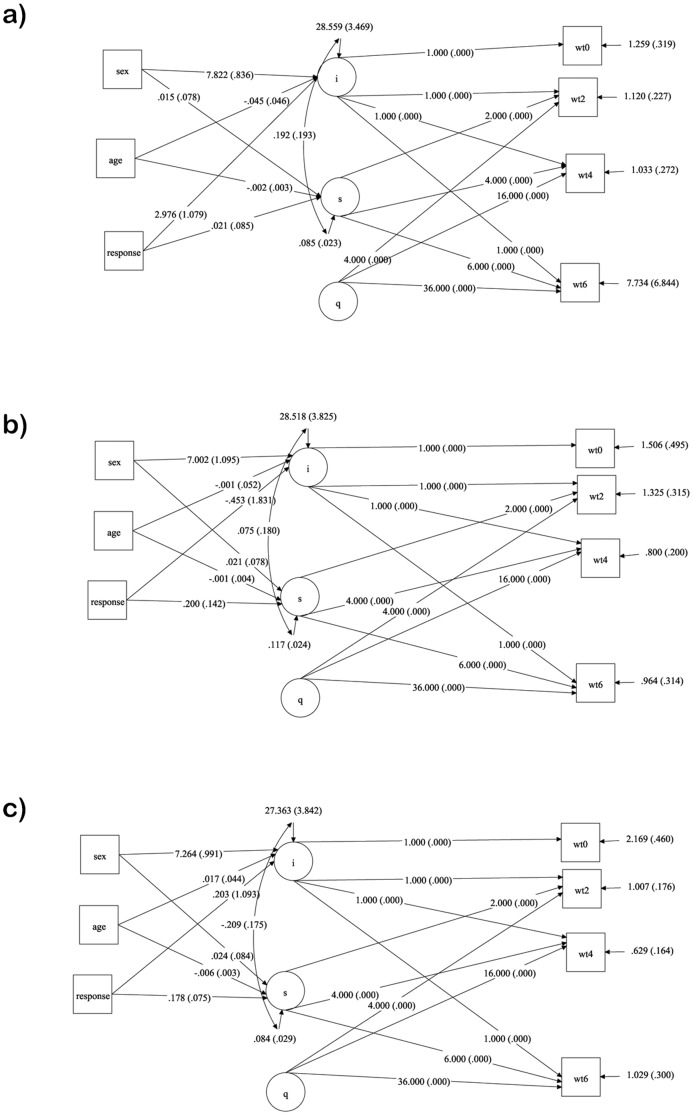
Quadratic latent growth models for three group comparisons of weight change in TB patients over time according to regimen with bootstrap sample of 1000 (a) regimen 1 (b) regimen 2 (c) regimen 3. Legend: i - intercept of quadratic latent growth model of weight change in TB patients. s - slope of quadratic latent growth model of weight changes in TB patients. q - quadratic factor of latent growth model of weight changes in TB patients. wt0 - weight of TB patients in kilograms at the initial stage of anti TB treatment. wt2 - weight of TB patients in kilograms at 2^nd^ month of anti TB treatment. wt4 - weight of TB patients in kilograms at 4^th^ month of anti TB treatment. wt6 - weight of TB patients in kilograms at 6^th^ month of anti TB treatment.

Because of RMSEA value of the model was higher, the conditional quadratic LGM was fitted by adding the covariates age, sex and response to explain the individual differences in initial mean and changes over time in the sample of 1000 using bootstrap method. The mean intercept, slope and parameter estimates for the multi groups LGM analysis of weight scores of the patients who received the three regimens are presented in Table 3. The fit indices for the model was improved (CFI  = 0.986, TLI  = 0.974, RMSEA  = 0.110) than the linear LGMs and the basic quadratic LGM. All the three groups showed a significant increase in weight scores while the increase was little higher for the group 3 (1.408, p<0.001) as compared to group1 (1.344, p<0.001) and group 2 (1.183, p<0.001). But a significant quadratic effect showed weights were sustained over time (Table 3). There were no group differences in intercept and slopes by assuming there was no variance for quadratic effect in the three groups. Gender differences were found to be statistically significant in the intercepts of three groups. The corresponding estimates of intercept on the covariate sex are 7.822 (S.E  = 0.836, p<0.001), 7.002 (S.E  = 1.095, p<0.001) and 7.264 (S.E  = 0.991, p<0.001) for group1, 2 and 3 respectively and no differences were observed in slopes. Hence, there were no differences in the change of weight scores across the three regimen groups of TB patients during the treatment period. There were no age effects on intercepts and slopes in the three groups except for the third group. The response was significantly associated with increase in the weight scores of first regimen group at the intercept level (2.976, S.E  = 1.079, p<0.01) and in the third regimen group at slope level (0.178, S.E  = 0.075, p<0.05). The path diagram of multiple group quadratic growth models for regimen 1, 2 and 3 are shown in Figure 5.

**Table 3:** Parameter Estimates of quadratic LGM.

Parameter/Fit	Group 1	Group 2	Group 3	Without group
Statistics	Estimate (S.E)	Estimate (S.E)	Estimate (S.E)	Estimate (S.E)
Regression weights				
Intercept (mean)	33.358 (1.612)*	35.892 (2.332)*	33.813 (1.633)*	34.161 (0.940)*
Slope (mean)	1.344 (0.129)*	1.183 (0.201)*	1.408 (0.140)*	1.345 (0.085)*
Quadratic (mean)	−0.115 (0.012)*	−0.112 (0.011)*	−0.126 (0.013)*	−0.126 (0.009)*
Intercept on				
Sex	7.822 (0.836)*	7.002 (1.095)*	7.264 (0.991)*	7.310 (0.569)*
Age	−0.045 (0.046)	−0.001 (0.052)	0.017 (0.044)	−0.006 (0.026)
Outcome	2.976 (1.079)^$^	−0.453 (1.831)	0.203 (1.093)	1.046 (0.078)
Slope on				
Sex	0.015 (0.078)	0.021 (0.078)	0.024 (0.084)	0.043 (0.053)
Age	−0.002 (0.003)	−0.001 (0.004)	−0.006 (0.003)^+^	−0.004 (0.002)^+^
Outcome	0.021 (0.085)	0.200 (0.142)	0.178 (0.075)^+^	0.138 (0.053)^$^
Variances				
Intercept	28.559 (3.469)*	28.518 (3.825)*	27.363 (3.842)*	28.202 (2.117)*
Slope	0.085 (0.023)*	0.117 (0.024)*	0.084 (0.029)^#^	0.102 (0.015)*
Covariances				
Slope with intercept	0.192 (0.193)	0.075 (0.180)	−0.209 (0.175)	0.098 (0.121)
R Square				
Wt0	0.970	0.963	0.945	0.962
Wt2	0.974	0.968	0.973	0.973
Wt4	0.977	0.981	0.983	0.980
Wt6	0.858	0.979	0.973	0.920
Intercept	0.305	0.281	0.263	0.278
Slope	0.005	0.024	0.105	0.032

*p<0.001, #p<0.005, +p<0.05, $p<0.01.

**Figure 6:**
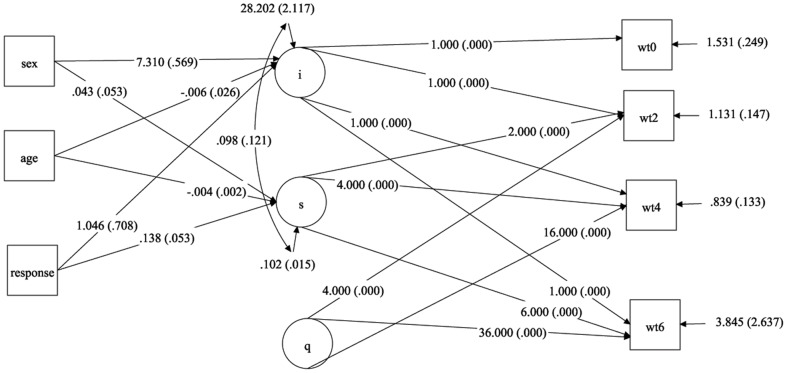
Quadratic growth model of weight change in TB patients over time with bootstrap sample of 1000. Legend: i - intercept of quadratic latent growth model of weight change in TB patients. s - slope of quadratic latent growth model of weight changes in TB patients. q - quadratic factor of latent growth model of weight changes in TB patients. wt0 - weight of TB patients in kilograms at the initial stage of anti TB treatment. wt2 - weight of TB patients in kilograms at 2^nd^ month of anti TB treatment. wt4 - weight of TB patients in kilograms at 4^th^ month of anti TB treatment. wt6 - weight of TB patients in kilograms at 6^th^ month of anti TB treatment.

A better fit was produced when the model was analysed with sample of 1000 using bootstrap method, by adding the covariates age, sex and response (CFI  = 0.991, TLI  = 0.983 and RMSEA  =  0.088). For this model, there was a significant increase in the weight (1.345, p<0.001) while a significant quadratic effect which indicated that weights were sustained over time. A statistically significant gender difference was identified in the change of weight over time on the latent factor intercept (7.310, S.E  = 0.569, p<0.001) but not in slope. The response was significantly associated with increase in weight scores of the patients over time at the slope level (0.138, S.E  = 0.053, p<0.01). Age of the patients was negatively associated with the latent factor slope (Table 3). Hence, the individual TB patients differ in their initial level of weight and their growth pattern. The RMSEA value of the model was very closer to the cut off level 0.08, the quadratic model have better fit statistics and satisfied the cut-off levels for CFI and TLI. The values of BIC and AIC were low compared to other linear LGM models and other quadratic models. The path diagram of the model is shown in Figure 6. In the study, when the sample was increased to 5000 and 10,000 using bootstrap method, similar kinds of results were obtained in linear LGM and quadratic LGM models without grouping and multiple group modeling.

## Discussion

Structural equation modeling is powerful tool for modeling LGM to analyse dynamic changes. LGM has several advantages in using longitudianl design than conventional techniques such as repeated analysis of variance (RM-ANOVA). One among the strengths of LGM is the use of latent repeated measures which is not used in other techniques of model trajectory [18]. LGM was very helpful in this study to identify a complete picture of a linear or nonlinear change of parameter estimations along with their co-variances. The main aim of the paper was to explore the LGM models in weight changes of the TB patients under different regimens. LGM and Quadratic LGM were fitted for the weight of TB patients using the initial stage of treatment as the baseline for assessing pattern of changes over time. The quadratic LGM without grouping was found to be better model than multiple group quadratic LGM for studing the changing pattern of weight of the TB patients.

A statistically significant gender difference was identified in the change of weight over time on the latent factor intercept but not in slope. But the age effect on slopes was having negative association with the wieght scores where as the response was significantly associated with increase in weight scores of the patients over time. Weight loss is one of the major symptoms along with other respiratory disease in patients with TB at the time of TB diagnosis [26], [27], [28], [29]. Once TB patients start to receive anti-TB treatment, there would be an increase in concomitant appetite and weight gain. When the infectivity symptoms are reduced, the weights are sustained over time. These may be probable reasons for the trend of weight of TB patients over time follows nonlinear.

There are many studies from our centre that have shown that the effect of gain in body weight on the treatment outcome. An epidemiological survey conducted in Tiruvallur district, south India reported that higher death rates of TB patients were associated with their initial loss of body weight (<35 kgs) [30] and cure of TB patients were associated with gain of weight during treatment period [10]. A study from Peru reported that patients with good outcome gained, on average, almost 1 kg compared to their baseline weight, whereas those with poor outcome lost 1 kg after adjusting for age, gender, type of TB, scheme of treatment, HIV status, and sputum variation during follow-up, after the first month of treatment [12]. Hao et al, 2013 [11], assessed the relationship between change in weight and anti TB treatment outcome. They found that new sputum smear positive TB patients with a successful treatment outcome gained an average of 2.6 kg during treatment. Patients with weight loss during the first two months of treatment were more likely to have an unsuccessful outcome than those without weight loss.

Our quadratic LGM model identified the trend of weight scores and the significance between the weight score and the treatment out come. Hence Quadratic LGM models are valuable tools to predict the changes in weight scores of TB patients that determine the progress or failure of the treatment. The data considered in this paper were limited and did not include other important assessments of TB diagnosis which are related to cure of the patients. The other extensions and applications of growth curve models include growth mixture models, piecewise growth curve models, modeling changes in latent variables and structured latent curve models need further research.

## Supporting Information

Appendix S1 **M** ***plus*** **commands used for analyses.** Legend: i - intercept of latent growth model of weight changes in TB patients. s - slope of latent growth model of weight changes in TB patients. wt0 - weight of TB patients in kilograms at the initial stage of anti TB treatment. wt2 - weight of TB patients in kilograms at 2^nd^ month of anti TB treatment. wt4 - weight of TB patients in kilograms at 4^th^ month of anti TB treatment. wt6 - weight of TB patients in kilograms at 6^th^ month of anti TB treatment.
(DOC)
