# Supplementary material for: Structural Equation Modeling of Latent Growth Curves of Weight Gain among Treated Tuberculosis Patients
Source: PLoS One. 2014 Mar 11;9(3):e91152. doi: 10.1371/journal.pone.0091152 (PMC3949979; doi:10.1371/journal.pone.0091152)
Supplement: Appendix S1 — M plus commands used for analyses. Legend: i - intercept of latent growth model of weight changes in TB patients. s - slope of latent growth model of weight changes in TB patients. wt0 - weight of TB patients in kilograms at the initial stage of anti TB treatment. wt2 - weight of TB patients in kilograms at 2nd month of anti TB treatment. wt4 - weight of TB patients in kilograms at 4th month of anti TB treatment. wt6 - weight of TB patients in kilograms at 6th month of anti TB treatment. (DOC) [file pone.0091152.s001.doc]

**Appendix S1 M*plus* commands used for analyses**

**Unconditional Linear LGM**

DATA: FILE IS weight.dat;

VARIABLE: NAMES ARE REGIMEN SEX AGE OUTCOME WT0 WT2 WT4 WT6;

USEVARIABLES ARE WT0 WT2 WT4 WT6;

MODEL: i s | WT0@0 WT2@2 WT4@4 WT6@6;

OUTPUT: stdyx tech1;

**Unconditional Quadratic LGM**

DATA: FILE IS weight.dat;

VARIABLE: NAMES ARE REGIMEN SEX AGE OUTCOME WT0 WT2 WT4 WT6;

USEVARIABLES ARE WT0 WT2 WT4 WT6;

MODEL: i s q | WT0@0 WT2@2 WT4@4 WT6@6;

q@0;

OUTPUT: stdyx tech1;

**Conditional Quadratic LGM by bootstrap method**

DATA: FILE IS weight.dat;

VARIABLE: NAMES ARE REGIMEN SEX AGE RESPONSE WT0 WT2 WT4 WT6;

USEVARIABLES ARE SEX AGE RESPONSE WT0 WT2 WT4 WT6;

ANALYSIS: BOOTSTRAP = 1000;

MODEL: i s q | WT0@0 WT2@2 WT4@4 WT6@6;

q@0;

i s ON SEX AGE RESPONSE;

OUTPUT: stdyx tech1;

**Unconditional Multi group Quadratic LGM by bootstrap method**

DATA: FILE IS weight.dat;

VARIABLE: NAMES ARE REGIMEN SEX AGE RESPONSE WT0 WT2 WT4 WT6;

USEVARIABLES ARE REGIMEN WT0 WT2 WT4 WT6;

GROUPING IS REGIMEN(1 = REGIMEN1 2 = REGIMEN2 3 = REGIMEN3);

ANALYSIS: BOOTSTRAP = 1000;

MODEL: i s q | WT0@0 WT2@2 WT4@4 WT6@6;

q@0;

OUTPUT: stdyx tech1;

**Conditional Multi group Quadratic LGM by bootstrap method**

DATA: FILE IS weight.dat;

VARIABLE: NAMES ARE REGIMEN SEX AGE RESPONSE WT0 WT2 WT4 WT6;

USEVARIABLES ARE REGIMEN SEX AGE RESPONSE WT0 WT2 WT4 WT6;

GROUPING IS REGIMEN(1 = REGIMEN1 2 = REGIMEN2 3 = REGIMEN3);

ANALYSIS: BOOTSTRAP = 1000;

MODEL: i s q | WT0@0 WT2@2 WT4@4 WT6@6;

q@0;

i s ON SEX AGE RESPONSE;

OUTPUT: stdyx tech1;
